# Efficacy and safety of stellate ganglion block for tinnitus: a systematic review and meta-analysis

**DOI:** 10.3389/fneur.2026.1766506

**Published:** 2026-01-20

**Authors:** Jiao Liang, He Ming, Qingzhu You, Han Xie, Jie Zhou, Qian Xiong

**Affiliations:** 1 College of Traditional Chinese Medicine, Chongqing Three Gorges Medical College, Chongqing, China; 2 Acupuncture and Moxibustion Department, People’s Hospital Affiliated to Chongqing Three Gorges Medical College, Chongqing, China

**Keywords:** clinical research, efficacy evaluation, meta-analysis, stellate ganglion block, tinnitus

## Abstract

**Purpose:**

This systematic review and meta-analysis evaluates the effectiveness and safety of stellate ganglion block (SGB) for tinnitus.

**Methods:**

A comprehensive systematic literature search was performed across four Chinese databases include China National Knowledge Infrastructure (CNKI), Wanfang, China Science and Technology Journal Database (VIP), and SinoMed. five English databases include PubMed, Cochrane Library, Embase, Ovid, and Web of Science to identify randomized controlled trials (RCTs) investigating the use of SGB for tinnitus treatment published before November 28, 2025. Searches were conducted in both Chinese and English. Following a rigorous screening process, meta-analyses were carried out using Stata 17.0 and RevMan 5.2.1 software. The study protocol was registered on PROSPERO (CRD420251242113).

**Results:**

A total of 11 randomized controlled trials comprising 915 patients were included in this study. Meta-analysis demonstrated that SGB combined other therapy was significantly more effective than the control group in treating tinnitus, with an overall effective rate (OR = 4.53, 95% CI [3.15, 6.53], *p* < 0.00001). In terms of functional improvement, SGB significantly reduced the THI (MD = −5.73, 95% CI [−6.10, −5.36], *p* < 0.00001) and the SAS (MD = −11.37, 95% CI [−12.46, −10.29], *p* < 0.00001). Hemodynamic assessments revealed a notable increase in basilar artery blood flow velocity following SGB treatment (Vs: MD = 5.60 cm/s, 95% CI [4.40, 6.80], *p* < 0.00001; Vd: MD = 4.26 cm/s, 95% CI [3.70, 4.83], *p* < 0.00001). Similarly, carotid artery blood flow velocity showed significant improvement (PSV: MD = 4.73 cm/s, 95% CI [3.26, 6.18], *p* < 0.00001; EDV: MD = 10.85 cm/s, 95% CI [6.02, 15.68], *p* < 0.0001).

**Conclusion:**

SGB combination therapy shows promise in managing tinnitus by improving effective rates, blood flow, THI and SAS scores. However, future large-scale, rigorous trials are essential to standardize treatment, address potential bias, and confirm long-term benefits.

**Systematic review registration:**

https://www.crd.york.ac.uk/PROSPERO, identifier CRD420251242113.

## Background

Tinnitus is a common auditory symptom with a high global prevalence that significantly impairs quality of life. Epidemiological data indicate that approximately 14.4% of adults are affected by tinnitus, with the prevalence rising to as high as 23.6% among elderly populations (1, 2). The impact of tinnitus extends far beyond the auditory system, often accompanying sleep disturbances, anxiety and depression, and even leading to cognitive decline and social withdrawal, posing a substantial disease burden on both individuals and society (3–5). At present, the pathophysiological mechanisms of tinnitus have not been fully elucidated, and there is a lack of curative treatments in clinical practice. Although mainstream interventions such as cognitive behavioral therapy and sound therapy have demonstrated certain efficacy, their widespread application remains limited due to practical constraints including a shortage of specialized healthcare professionals and the high cost of equipment (6).

SGB is a minimally invasive interventional technique targeting the cervical sympathetic ganglion. Its core mechanism involves blocking sympathetic nerve signals to restore balance in the autonomic nervous system. Based on this mechanism, SGB has long been used in the treatment of various pain-related conditions and neurological disorders, demonstrating considerable clinical potential. Notably, in recent years, this technique has also been introduced into the management of tinnitus, gradually gaining attention among researchers and clinicians (7, 8). At the operational level, SGB typically involves injecting local anesthetic at specific anatomical sites in the neck. By suppressing excessive sympathetic excitation, improving blood perfusion around the ear and intracranial regions, and modulating central neural plasticity, it exerts multi-pathway effects that alleviate tinnitus symptoms (9, 10).

The application of SGB in tinnitus treatment has a relatively long history. To date, multiple clinical studies have been conducted, including randomized controlled trials, before-after studies, and case series. The results of these studies suggest that SGB may be an effective therapeutic approach for tinnitus (11–13). For instance, one study demonstrated that patients receiving SGB intervention showed significant improvements in both the THI (tinnitus handicap inventory) score and anxiety scores, further supporting the potential clinical value of this method in alleviating tinnitus-related symptoms (14).

However, there is currently a lack of systematic reviews and meta-analyses to quantitatively synthesize this body of evidence, making it difficult to draw definitive conclusions regarding the efficacy of SGB in the treatment of tinnitus. Therefore, this study aims to conduct a systematic review and meta-analysis to comprehensively evaluate the overall effectiveness and safety of SGB for tinnitus. The findings are expected to provide high level evidence based support for the clinical application of SGB in tinnitus management and to guide the direction of future high quality research.

## Method

### Search strategy

We searched nine databases, including four Chinese databases (CNKI, WanFang, VIP and SinoMed), five English databases (PubMed, Cochrane Library, Embase, Ovid, and Web of Science) and a supplementary search was conducted by screening the reference lists of all included articles. A systematic search was conducted in these databases for literature on the treatment of “tinnitus” using “stellate ganglion block.” The strategy was adapted per database. For the Chinese databases (CNKI, WanFang, VIP, and SinoMed), we utilized the built-in subject headings (where applicable) in combination with searches in the title, abstract, and keyword fields, while also incorporating synonym expansion to enhance recall. For the English search, MeSH terms combined with title/abstract/keyword searches were employed, terms such as “Stellate Ganglion,” “Cervicothoracic Ganglion,” and “Tinnitus,” were used, As illustrated by the Web of Science example, the Topic (TS) field was employed to search for terms within titles, abstracts, and keywords. For exsample: (TS = (“stellate ganglion” NEAR/2 (block* OR inject* OR anesth*)) OR TS = (“cervicothoracic ganglion” NEAR/2 (block* OR inject*)) OR TS = (“stellate ganglion block”) OR TS = (“stellate block”) OR TS = (“stellate ganglion injection”) OR TS = (SGB) OR TI = (“stellate ganglion block”)) AND (TS = (tinnitus) OR TS = (“ringing in the ears”) OR TS = (“ear noise*”) OR TS = (“phantom sound*”) OR TS = (“subjective tinnitus”) OR TI = (tinnitus)). The search period covered from the inception of each database until November 28, 2025. The comprehensive search syntax and strategy employed for each individual database are provided in Supplementary Data Sheet S1.

### Inclusion criteria

*Study design and population*: Parallel-designed RCTs involving patients diagnosed with subjective tinnitus.

*Interventions*: Experimental group: Must receive SGB as the primary or sole intervention. Control group: Permissible controls include pharmacological therapy, sham injection (e.g., normal saline injection), no treatment, or other therapies (e.g., cognitive behavioral therapy, sound therapy). Examples of valid comparisons: SGB vs. sham injection, SGB vs. other therapies, SGB combined with baseline treatment vs. baseline treatment alone.

*Outcome measures*: Studies must report at least one of the following primary or secondary outcomes: (1) Clinical total effective rate. (2) THI score. (3) Self-rating Anxiety Scale (SAS) score or other validated psychometric tools. (4) Incidence of adverse events.

*Publication type*: Full-text articles published in peer-reviewed journals.

*Language restriction*: Articles published in either Chinese or English.

### Exclusion criteria

*Intervention*: Studies in which the experimental group did not receive SGB as a core intervention.

*Population*: Studies including patients with objective tinnitus, pulsatile tinnitus, or tinnitus resulting from specific otological surgeries.

*Study design*: Non-randomized studies, case reports, conference Papers, graduation thesis, study protocols, reviews, mechanistic studies, or studies with republished data.

*Data integrity*: Studies with missing critical data, inconsistent statistical reporting, or outcomes that cannot be extracted or analyzed.

### Data collection

Two researchers (JL and JZ) independently screened the literature. First, they performed a preliminary screening based on titles and abstracts. Subsequently, they evaluated the full texts of potentially eligible studies. Throughout this process, publication types deemed irrelevant were excluded, such as reviews, animal studies, case reports, and non-randomized controlled trials. Any disagreements that arose during the screening process were resolved through consultation with a third reviewer (QX) until a consensus was reached.

The data extraction process included the following elements:

1. *Basic study information*: first author, publication year, and diagnostic criteria.
2. *Study characteristics*: sample size, gender distribution, age, disease duration, inter-group comparison method, control group interventions, ethical approval status, and SGB procedural details (e.g., specific drugs used, number of sessions, follow-up status).
3. *Outcome measures*: Primary and secondary outcomes reported in the studies.

### Quality assessment

The methodological quality of the included studies was assessed according to the criteria outlined in the Cochrane Handbook for Systematic Reviews. The evaluation focused on the following seven domains of bias risk: randomization method, allocation concealment, blinding, outcome assessment bias, completeness of outcome data, selective reporting, and other potential sources of bias. Each domain was independently rated as “low risk,” “high risk,” or “unclear risk.” A risk of bias graph was generated to summarize the assessments. Two researchers (JL and JZ) performed the evaluation independently, followed by cross checking. Any disagreements were resolved through discussion with a third researcher (QX) until a consensus was reached.

### Statistical analysis

Meta-analysis was performed using RevMan 5.2 and Stata 17.0 software. Outcome measures included the effective rate, THI score, hemodynamic parameters of the carotid artery (peak systolic velocity PSV and end-diastolic velocity EDV), and hemodynamic parameters of the vertebrobasilar artery (peak systolic velocity, end-diastolic velocity). Forest plots were generated for these outcomes. Heterogeneity was assessed using the *I*^2^ statistic, with an *I*^2^ value greater than 50% considered indicative of substantial heterogeneity. A random effects model was applied for analyses with significant heterogeneity (*I*^2^ > 50%); otherwise, a fixed effects model was used. Pooled effect sizes were reported with 95% confidence intervals (CI), and a *p* < 0.05 was considered statistically significant.

If substantial heterogeneity was detected, subgroup analyses were conducted based on control group interventions, the number of SGB injections, and technical details of the SGB procedure to explore potential sources of heterogeneity. Sensitivity analysis was carried out using the leave-one-out method to examine the robustness of the results. Finally, funnel plots were used to evaluate potential publication bias.

### Search results

**Figure 1:**
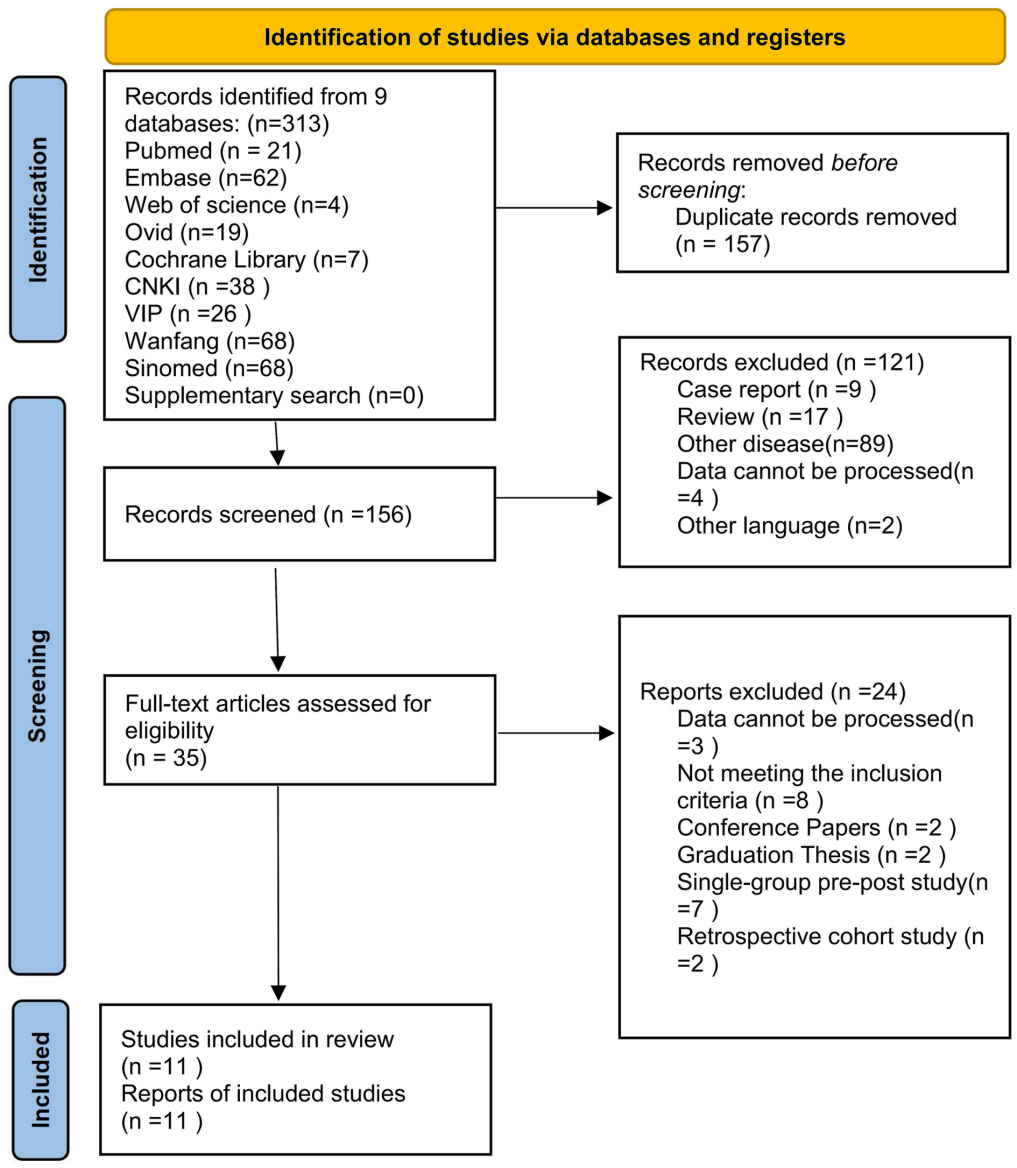
Flow diagram of literature screening and selection outcomes.

An initial search of Chinese and international databases yielded 313 relevant records. After removing 157 duplicates using EndNote X7 software, 156 records remained. Two researchers independently screened the titles and abstracts of these records and excluded 121 irrelevant publications, which included 9 case reports, 17 reviews, and 89 articles focusing on other diseases. Subsequently, full-text assessments were conducted on the remaining 35 articles. Among these, 3 were excluded due to unavailability of the full text, 8 did not meet the inclusion criteria, and 11 were excluded based on study design (2 conference papers, 2 dissertations, 7 before-after studies, and 2 retrospective studies). Finally, 11 clinical studies that satisfied all eligibility criteria were included in the meta-analysis. The detailed screening process is illustrated in Figure 1.

### Characteristics of the included studies

Based on the predefined inclusion and exclusion criteria, 11 studies published between 2012 and 2025 were included in this review. All studies were RCTs, involving a total of 915 patients with subjective tinnitus. Among them, 459 patients were assigned to the experimental group (SGB combined with medication) and 456 to the control group (medication alone). The studies varied in their focus on population characteristics: Du (15) focused on elderly patients, Li (16) mainly included middle-aged patients, Wang B (17) and Qian (14) targeted chronic tinnitus, Wang R (18) focused on acute tinnitus, and Wang (19) enrolled patients with tinnitus accompanied by depressive symptoms.

### Interventions and outcome measures

In all studies, the experimental group received SGB combined with medication, while the control group received medication alone, with specific drug regimens differing across studies. Six studies reported obtaining ethical approval (14, 16–20).

Four studies (16, 17, 19, 20) reported follow-up durations, covering a total of 13 outcome categories. The most frequently reported outcome was the overall effective rate, which appeared in all 11 studies (14–24). The distribution of other outcome measures was as follows: SAS score: 7 studies (14–16, 18, 21–23), THI score: 5 studies (14, 17, 21–23), Carotid artery blood flow velocity: 2 studies (21, 23), Basilar artery blood flow velocity:3studies (20, 21, 24), Adverse reactions: 6 studies (15–18, 20, 24), Satisfaction rate: 2 studies (18, 19), Tinnitus loudness and dominant frequency: 1 study (17), Pittsburgh Sleep Quality Index (PSQI) score: 1 study (18). The basic characteristics of the included studies are summarized in Tables 1, 2.

**Table 1:** Basic characteristics of included literature.

Author year	Diagnosis	Male/Female		Age (years)		Course of disease		Tinnitus severity (SGB vs. C)	Ethics approval
		SGB	C	SGB	C	SGB	C		
Du 2020 (15)	elderly tinnitus	28/22	31/19	70.64 ± 3.27	69.45 ± 3.18	8.57 ± 2.89(m)	8.62 ± 2.74(m)	III:IV:V = 16:25:9 vs. 14:28:8	N
Xie 2022 (24)	tinnitus	25/15	26/14	34–70, 52.41 ± 3.31	35–72, 52.39 ± 3.28	1-18(m), 7.61 ± 1.41(m)	2-19(m), 7.59 ± 1.38(m)	III:IV:V = 11:13:16 vs. 10:12:18	N
Li 2012 (16)	middle-aged tinnitus	NR	NR	40–60		0.5–4 (y)		unilateral	Y
Shen 2015 (21)	tinnitus	31/27		40–60, 51.7 ± 5.4		3(m)-3(y), 1.4 ± 0.4(y)		unilateral	N
Liu 2016 (22)	tinnitus	NR	NR	NR	NR	NR		NR	N
Yuan 2018 (20)	tinnitus	30/20	32/18	61.9 ± 10.3	61.6 ± 10.3	1–18 (m), 8.6 ± 3.6 (m)	1–18 (m), 8.32 ± 1.57 (m)	III:IV:V = 14:29:7 vs. 16:28:6	Y
Yuan et 2018 (23)	tinnitus	18/15	18/14	41–60, 54.52 ± 5.87	40–60, 54.28 ± 5.69	NR		III:IV:V = 14:29:7 vs. 16:28:6	N
Wang 2019 (19)	tinnitus with depression	38/22		36–65, 45.7		<3 (m)		NR	Y
Wang 2024 (17)	chronic tinnitus	12/28	19/21	53.25 ± 11.2	49.71 ± 9.27	2.01 ± 0.11(y)	1.92 ± 0.27(y)	L:R = 19:21 vs. 25:15	Y
Wang R 2025 (18)	acute tinnitus	19/27	20/26	55.81 ± 2.92	55.42 ± 3.22	35.63 ± 18.11(d)	39.41 ± 16.92(d)	L:R:B = 19:25:2 vs. 19:23:4	Y
Qian 2025 (14)	chronic tinnitus	19/21	22/18	44 ± 5	43 ± 6	8.3 ± 0.6(m)	8.2 ± 0.8(m)	NR	Y

NR, not report; C, control group; d, day; m, month; y, year; L, left; R, right; B, bilateral; N, no; Y, yes.

**Table 2:** Basic characteristics of SGB detailed message.

Author year	Injection side	Guidance	Comparison	SGB regimen	Control regimen	Outcomes	Follow-up
Du 2020 (15)	NR	blind	SGB + C vs. C	1.7 mL 2% lidocaine+5.2 mL saline, qd*12 times	(mecobalamin 0.5 mg po tid + VB12 500 mg im qd) * 30d	(1)(2)(3)	NR
Xie 2022 (24)	NR	blind	SGB + C vs. C	3 mL lidocaine+3 mL saline, qd*12 times	(VB12 500 mg im qd + Ermen Tinghui auricular inj. qd + mecobalamin inj. qd) * 30d	(1)(3)(4)(5)	NR
Li 2012 (16)	NR	blind	SGB + C vs. C	2 mL 2% lidocaine+6 mL saline, q4d* 4–6 times	mecobalamin 0.5 mg po tid	(1)(2)(3)	1 month SGB (2 lost to follow-up, 2 withdrew) vs. C (2 withdrew)
Shen 2015 (21)	affected side	blind	SGB + C vs. C	2 mL 2% lidocaine+8 mL saline, q4d*4–6 times	(mecobalamin 0.5 mg + ATP 40 mg + VB1 20 mg + betahistine 6 mg) po, tid * 28d	(1)(2)(4)(5)(6)(7)(8)	NR
Liu 2016 (22)	affected side	blind	SGB + C vs. C	2 mL 2% lidocaine+8 mL saline, q4d*4–6 times	((mecobalamin 0.5 mg + citicoline 200 mg + VB120 mg + betahistine 6 mg) po, tid) * 28d	(1)(2)(8)	NR
Yuan 2018 (20)	affected side	blind	SGB + C vs. C	3.5 mL 1% lidocaine+3.5 mL saline qd*12 times	((mecobalamin 0.5 mg im + Ermen Tinghui auricular inj. + VB12, im) qd) * 30d	(1)(3)(4)(5)(9)	6 month Recurrence: SGB (1/48) vs. C (7/42)
Yuan et 2018 (23)	NR	blind	SGB + C vs. C	2 mL 2% lidocaine+8 mL saline, q4d*4–6 times	((mecobalamin 0.5 mg + citicoline 200 mg + VB120 mg + betahistine 6 mg) po, tid) *28d	(1)(2)(6)(7)(8)	NR
Wang 2019 (19)	NR	blind	SGB + C vs. C	10 mL 1% lidocaine qd*10 times	(alprostadil 10 Î¼g + Ginkgo 70 mg + dexamethasone 10 mg (after 3 days 5 mg, 3 days) + (flupentixol-melitracen, po + sound therapy)) * 10d	(1)(10)	6 month
Wang 2024 (17)	alternating sides	blind	SGB + C vs. C	4 mL 1% lidocaine + 1 mL mecobalamin injection, qd*10 times	(vincamine 30 mg bid + mecobalamin 0.5 mg tid) po * 10d	(1)(3)(8)(11)(12)	6 month
Wang R 2025 (18)	affected side	ultrasound	SGB + C vs. C	5 mL 0.2% ropivacaine, qd*7 times	(mecobalamin 0.5 mg + Ginkgo 0.19 g + Wuling 0.99 g) po, tid * 2w	(1)(2)(3)(10)(13)	NR
Qian 2025 (14)	affected side	ultrasound	SGB + C vs. C	5 mL 0.67% lidocaine, qd* 6times	(5 mL 2% lidocaine + 10 mL saline) iv gtt, qd * 10d	(1)(2)(8)	NR

Outcomes: (1) effective rate, (2) SAS (Self-rating Anxiety Scale), (3) Adverse Reactions, (4) Vs (Basilar Artery Blood Flow End-Diastolic Velocity); (5) Vd (Basilar Artery End-Diastolic Velocity); (6) PSV (Carotid Artery Peak Systolic Velocity); (7) EDV (Carotid Artery End-Diastolic Velocity); (8) THI (Tinnitus Handicap Inventory); (9) Recurrence Rate, (10) Patient Satisfaction, (11) Tinnitus Loudness; (12) Tinnitus Dominant Frequency; (13) PSQI (Pittsburgh Sleep Quality Index).

NR, Not Report; po, per os; im, intramuscular; inj, injection; iv gtt, Intravenous drip; qd, quaque die (once a day); bid, bis in die (twice a day); tid, ter in die (three times a day); d, day; w, week.

### Quality assessment

**Figure 2:**
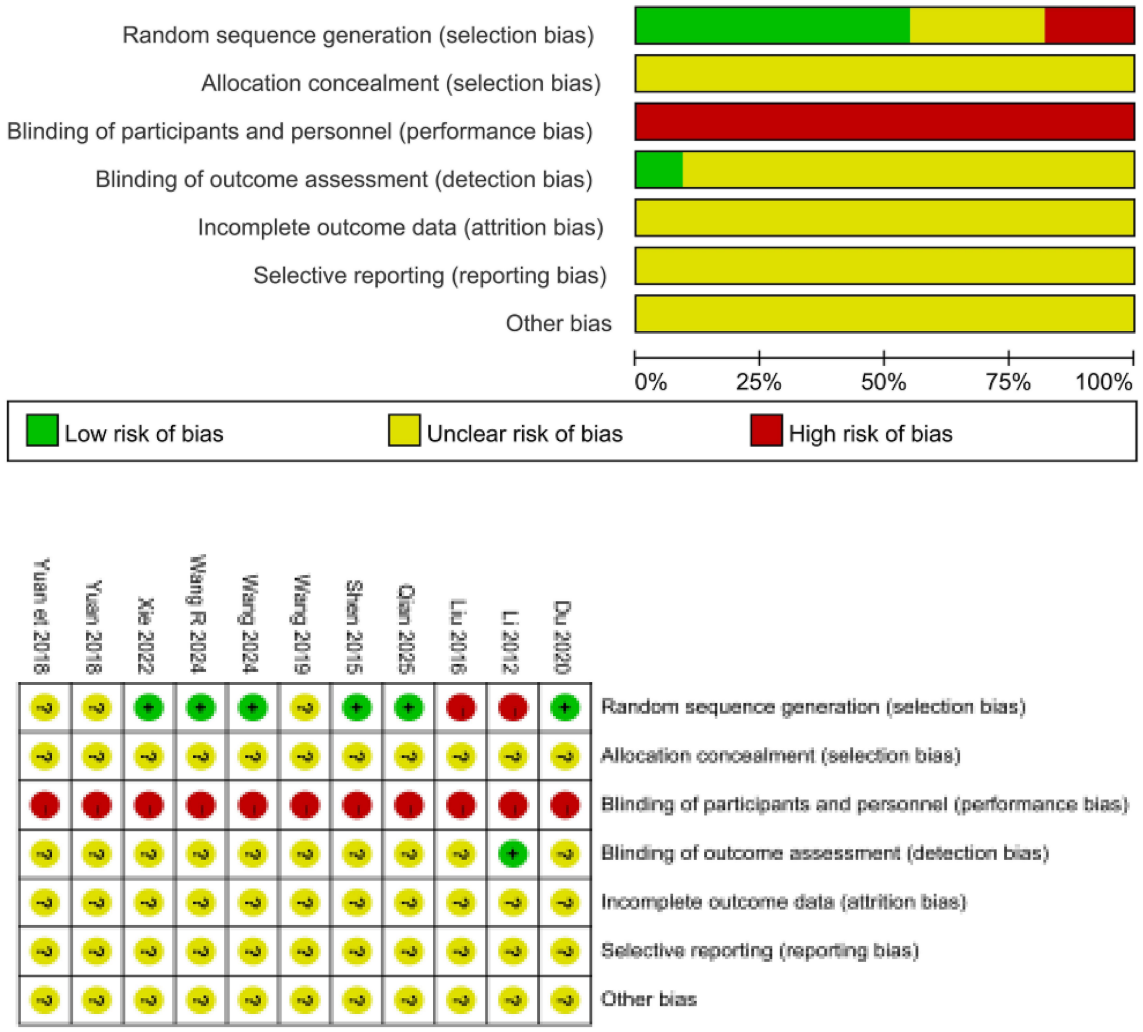
The figure represents the risk of bias assessment for the studies.

Regarding randomization methods, seven studies (14, 15, 17, 18, 20, 21, 23) used random number generation; one study (24) employed a two-color lottery method; two studies (12, 16) only mentioned “random allocation” without describing the specific method; and one study (19) did not explain the randomization procedure. In terms of blinding, one study (16) adopted a single-blind design for group assignment. Four studies (16, 17, 19, 20) reported follow-up outcomes, and six studies (15–18, 20, 24) reported adverse reactions. Additionally, one study (16) documented dropout cases, while all outcome measures were fully reported across the included studies. The detailed results of the methodological quality assessment are presented in Figure 2.

### Total effective rate

**Figure 3:**
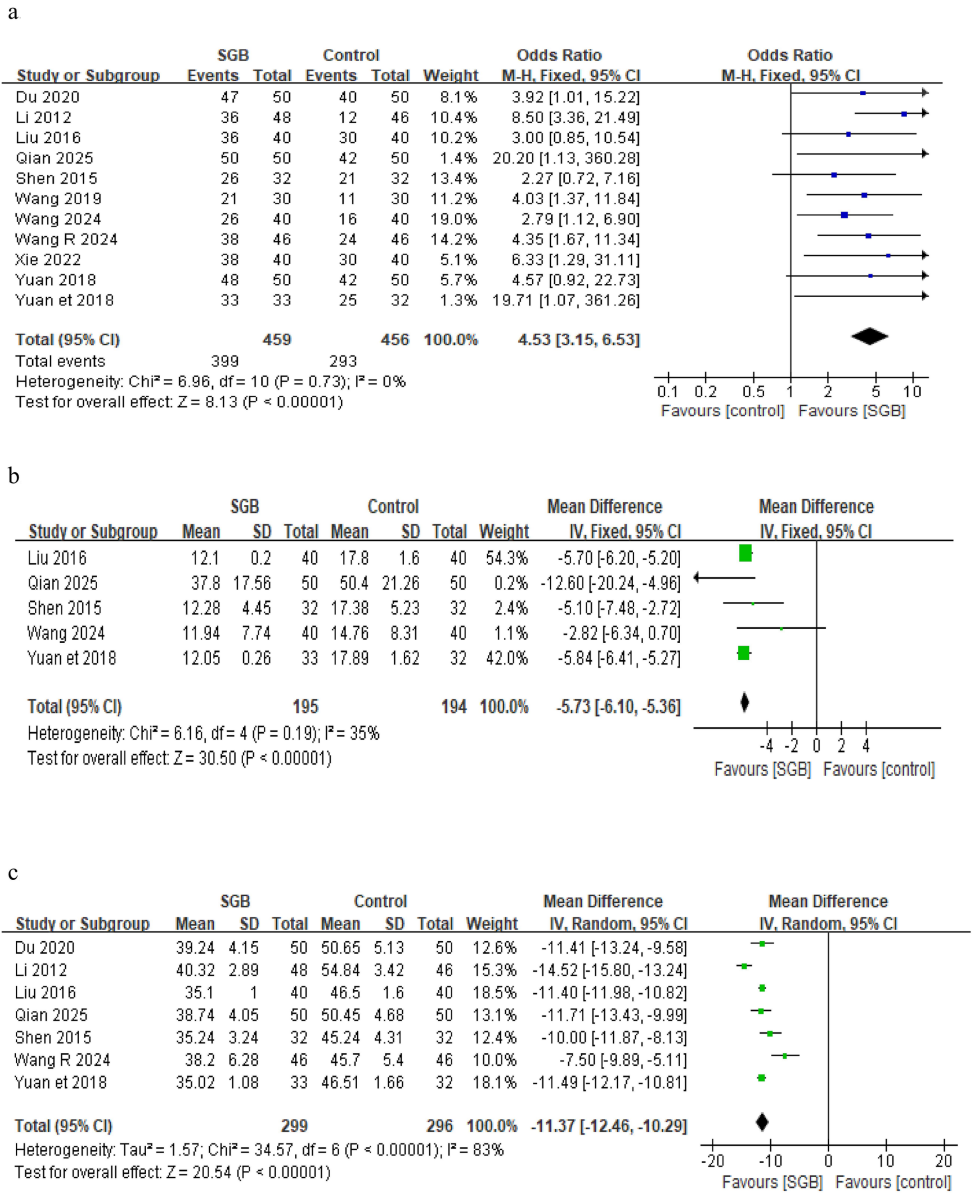
The figure represents a forest plot of total effective rate **(a)**, forest plot of THI scores **(b)**, forest plot of SAS scores **(c)**.

All 11 included studies reported the total effective rate. The heterogeneity test indicated low heterogeneity among the studies (*p* = 0.73, *I*^2^ = 0%), so a fixed effects model was used for the pooled analysis. The results showed a statistically significant difference between the groups (OR = 4.53, 95% CI [3.15, 6.53], *p* < 0.00001), indicating that the total effective rate in the SGB combined with medication group was significantly higher than that in the medication alone control group. Detailed results are shown in Figure 3a.

### THI score

A total of five studies (14, 17, 21–23) reported the THI score, involving 289 patients, with 195 in the experimental group (SGB combined therapy) and 194 in the control group. Heterogeneity testing indicated low heterogeneity among the studies (*p* = 0.19, *I*^2^ = 35%); therefore, a fixed effects model was used for the pooled analysis. The results showed that compared with the control group, SGB combined therapy significantly reduced the THI score by a mean difference of approximately 5.73 points (MD = –5.73, 95% CI [−6.10, −5.36], *p* < 0.00001), and this finding was highly consistent across all five included studies. The detailed forest plot is presented in Figure 3b.

### SAS score

A total of 7 studies (14–16, 18, 21–23) reported the SAS score, involving 595 patients, with 299 in the experimental group (SGB combined therapy) and 296 in the control group. Heterogeneity testing indicated high heterogeneity across the studies (*p* < 0.00001, *I*^2^ = 83%); therefore, a random effects model was used for the pooled analysis. The results showed that compared with the control group, SGB combined therapy significantly reduced the SAS score (MD = –11.37, 95% CI [−12.46, −10.29], *p* < 0.00001), the detailed forest plot is presented in Figure 3c.

### Basilar artery blood flow velocity

**Figure 4:**
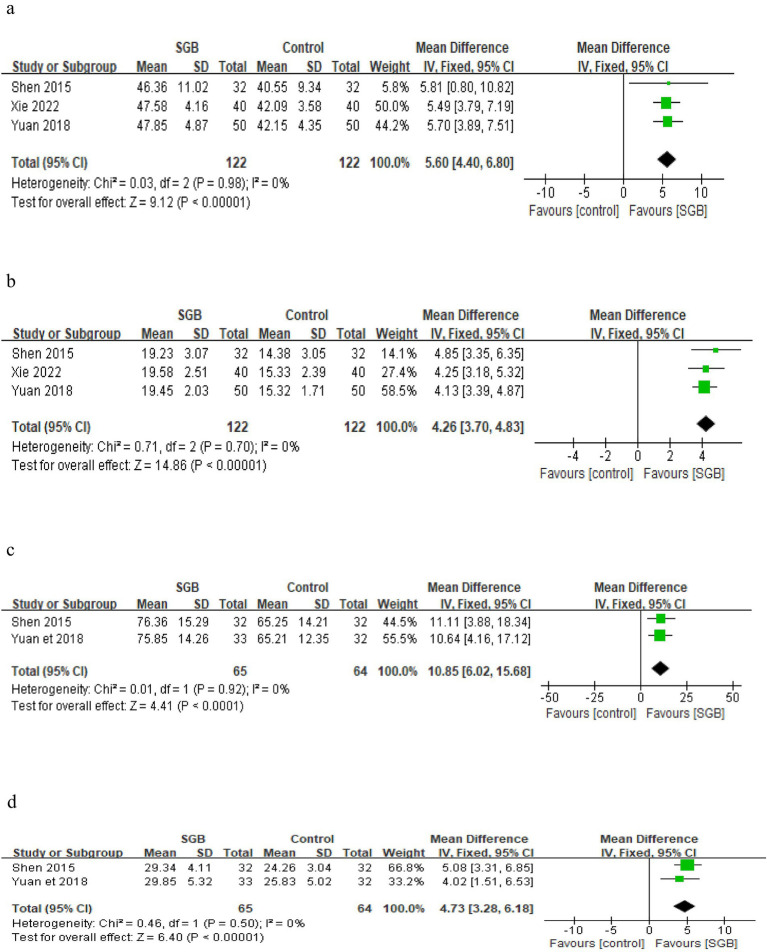
The figure represents a forest plot of Vs **(a)**, forest plot of Vd **(b)**, forest plot of PSV **(c)**, forest plot of EDV **(d)**.

A total of 3 studies (20, 21, 24) reported the peak systolic velocity (Vs) and end-diastolic velocity (Vd) of the basilar artery, involving 244 patients, with 122 in the experimental group and 122 in the control group. Heterogeneity testing indicated low heterogeneity among the studies (*I*^2^ = 0%); therefore, a fixed effects model was used for the pooled analysis. The results showed that compared with the control group, SGB combined therapy consistently and significantly increased the Vs value (MD = 5.60 cm/s, 95% CI [4.40, 6.80], *p* < 0.00001), suggesting improved perfusion in the vertebrobasilar artery system. Simultaneously, the Vd value was also significantly increased (MD = 4.26 cm/s, 95% CI [3.70, 4.83], *p* < 0.00001), reflecting reflexive dilation of the vascular system following treatment. Detailed forest plots for Vs and Vd are shown in Figures 4a,b.

### Carotid artery blood flow velocity

A total of 2 studies (21, 23) reported the peak systolic velocity (PSV) and end-diastolic velocity (EDV) of the carotid artery, with a cumulative sample size of 129 patients, including 65 in the experimental group (SGB combined therapy) and 64 in the control group. Heterogeneity testing indicated low heterogeneity among the studies (*I*^2^ = 0%); therefore, a fixed-effects model was used for the pooled analysis.

The results showed that, compared with the control group, SGB combined therapy consistently and significantly increased the PSV value (MD = 4.73 cm/s, 95% CI [3.26, 6.18], *p* < 0.00001), suggesting enhanced systolic blood flow perfusion in the carotid artery system. Meanwhile, the EDV value was also significantly increased (MD = 10.85 cm/s, 95% CI [6.02, 15.68], *p* < 0.0001), reflecting improved vascular diastolic function and indicating an overall increase in perfusion in the carotid artery system. Detailed forest plots for PSV and EDV are shown in Figures 4c,d.

### Adverse reactions

A total of 6 trials (15–18, 20, 24) reported adverse reactions. These mainly included dizziness (18), nausea and vomiting (15, 18, 24), palpitations (16), hoarseness (18, 20), throat discomfort (15, 24), and choking cough (18), among which nausea and vomiting were the most frequently reported, occurring 15 times in the SGB group and 13 times in the control group. The highest incidence of adverse events was recorded in Wang (18), affecting 29 out of 46 patients. All reported adverse events were mild and improved after observation or symptomatic treatment. These results indicate that although SGB carries certain risks of adverse reactions, its overall safety profile in tinnitus patients remains relatively favorable.

### Publication bias analysis

**Figure 5:**
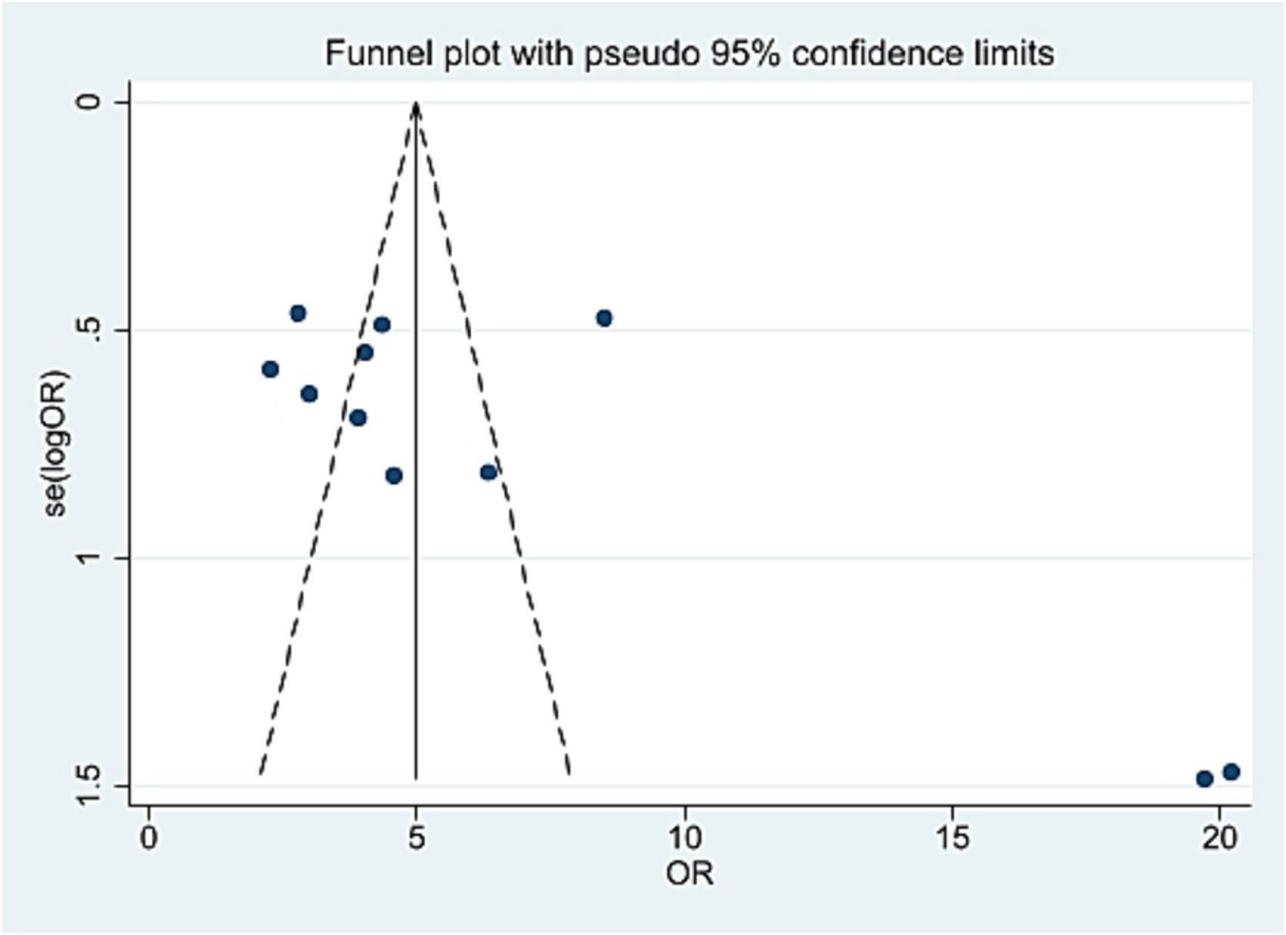
The figure represents the funnel plot.

**Figure 6:**
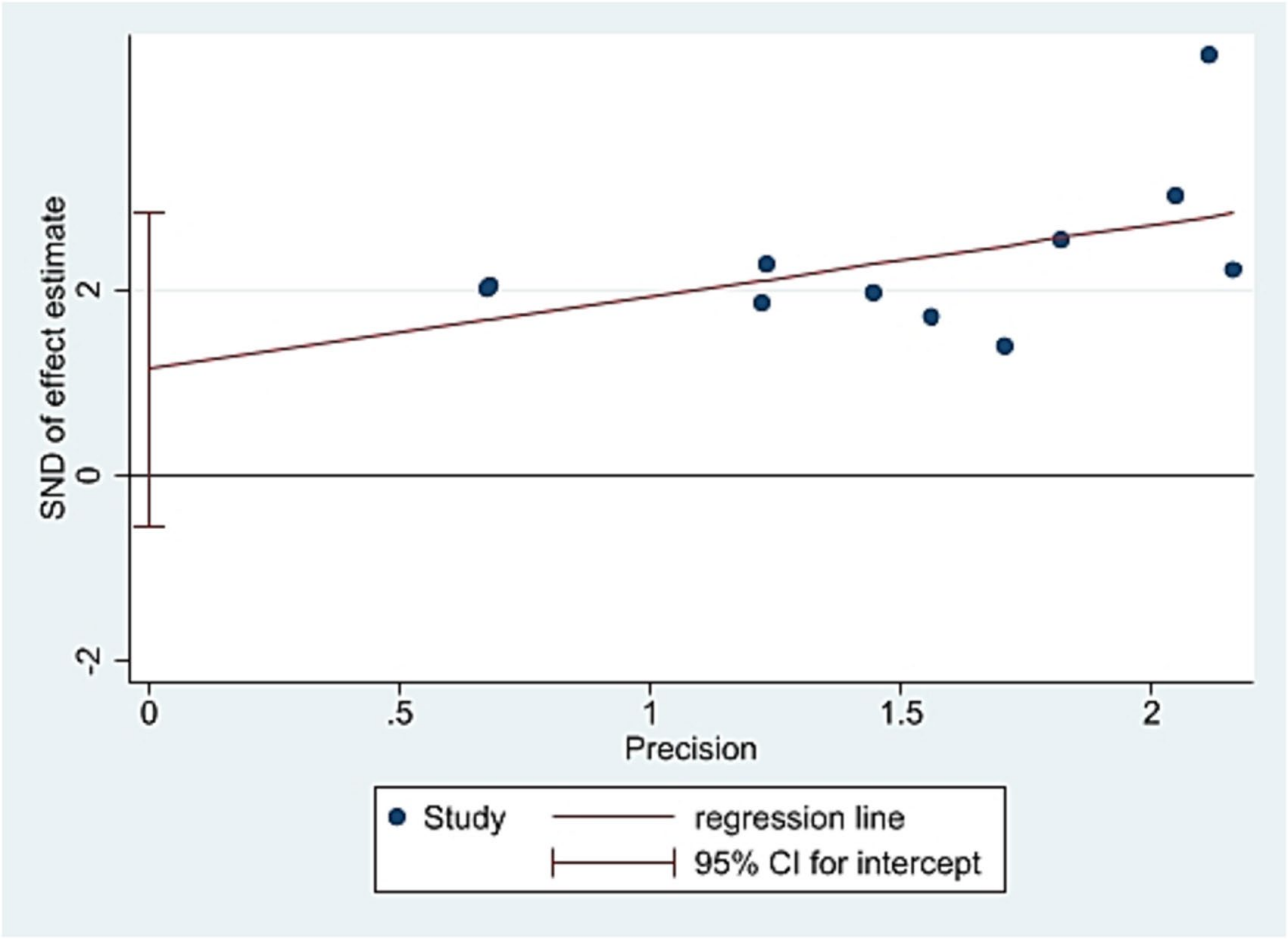
The figure represents the Egger’s test.

An inverted funnel plot was used to assess publication bias across the 11 included studies reporting the overall effective rate. The shape of the funnel plot showed asymmetry, suggesting the possible presence of publication bias (Figure 5). However, Egger’s linear regression test (*p* = 0.159, 95% CI [−0.54, 2.83]) indicated no statistical significance, demonstrating that although visual asymmetry was observed, no significant publication bias was detected statistically (Figure 6).

### Subgroups analysis and sensitivity analysis

**Figure 7:**
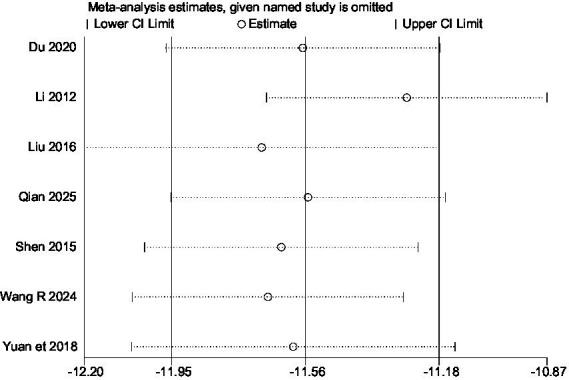
The figure represents the sensitivity analysis.

Regarding the high heterogeneity observed in SAS scores, we further conducted subgroup analyses based on the number of SGB injections and the use of ultrasound guidance. The *I*^2^ > 50%, and no clear source of heterogeneity could be identified (the supplement 2 section of Supplementary Data Sheet 1). Subsequently, a sensitivity analysis (Figure 7) showed that the pooled effect size did not change in direction, indicating that the main conclusions of the analysis are relatively robust.

## Discussion

This systematic review and meta-analysis confirmed that SGB combined with conventional therapy significantly improves the effective rate of tinnitus treatment, reduces tinnitus-related distress, and alleviates anxiety, with an acceptable safety profile. Specifically, the combined therapy increased the effective rate to 4.5 times that of conventional therapy (OR = 4.53, *I*^2^ = 0%), and significantly lowered the THI score (MD = −5.73), with highly consistent evidence across studies. Furthermore, SGB demonstrated clear improvement in patients’ anxiety status (*p* < 0.00001), indicating its value as a comprehensive psychosomatic intervention.

The therapeutic effects described above may originate from the multi-target integrative mechanism of SGB. First, the objective hemodynamic improvements revealed by the meta-analysis (increased PSV and EDV in the carotid artery, and elevated Vs and Vd in the basilar artery) provide key evidence, indicating that SGB alleviates vascular spasms by blocking sympathetic over-excitation, thereby improving the ischemic state of the inner ear and auditory centers (15, 25, 26). Subsequently, these improvements in carotid and basilar artery blood flow, combined with sympathetic inhibition, collectively modulate the central nervous system: not only supplying oxygen to neural tissues but also reducing the excitability of the locus coeruleus-noradrenergic system. This suppresses central sensitization in the auditory pathway, promotes normal neural plasticity, and attenuates tinnitus perception (27, 28).

Finally, through the “autonomic nervous system resetting” effect (inhibiting sympathetic activity while enhancing parasympathetic activity), SGB restores autonomic balance, thereby suppressing excessive responses in limbic system structures such as the amygdala. This alleviates tinnitus-related anxiety and stress responses, breaking the vicious cycle of “tinnitus-emotion-worsening tinnitus” (29). Together, these mechanisms form a complete pathway from peripheral circulation improvement to central neural regulation, and further to emotional circuit intervention.

Despite the clear therapeutic benefits, the following issues require attention in clinical application:

1. *Heterogeneity analysis*: The anxiety-related outcomes exhibited high heterogeneity (*I*^2^ = 83%). Existing subgroup analyses (e.g., number of SGB, use of ultrasound guidance) failed to fully explain its source. This suggests that future studies should conduct more detailed evaluation and reporting of patients’ baseline emotional status and combination treatment regimens.
2. *Safety and technical optimization*: Transient adverse reactions such as dizziness and hoarseness are common but generally mild and manageable. However, serious complications have been reported, such as the spread of local anesthetic into the subarachnoid space, which may lead to coma or even life-threatening conditions. To improve safety and treatment precision, ultrasound-guided SGB is recommended. This technique provides real-time visual feedback, reduces complications, and enhances the accuracy of drug distribution (14, 18, 30).
3. *Study limitations*: First, heterogeneity was observed in the definition of the primary outcome, the “total effective rate,” across the included studies. Among the 11 trials, three (15, 17, 18) applied the Liu (31) criteria, six (16, 19–23) used the Liu (32) criteria, one (14) employed the Grundfast KM (33) criteria, and one (24) study did not specify a standard but described an identical classification to Liu (32).

Specifically, the Liu (31) criteria defined outcomes as: clinical cure (complete disappearance of tinnitus), marked effect (reduction in tinnitus severity by ≥2 grades), effective (reduction by one grade), and ineffective (no change). The Liu (32) version added the requirement of “resolution of accompanying symptoms and no recurrence within a 1-month follow-up” for clinical cure, while the definitions for the other categories remained unchanged. The Grundfast KM (33) criteria, although using different terminology, followed the same core principle of grading improvement based on the reduction in tinnitus severity levels.

While all studies calculated the “total effective rate” consistently as the proportion of patients showing any positive improvement (i.e., clinical cure + marked effect + effective)—thus providing a methodological rationale for pooling—these definitional nuances remain a potential source of clinical and statistical heterogeneity.

Second, this study was limited by the quality of the original included trials. Some studies had deficiencies in randomization and blinding design. In addition, specific SGB operation parameters (e.g., drug dosage, treatment frequency) were not standardized, which may affect the accurate evaluation of efficacy. Furthermore, the lack of long-term follow-up data means the sustained efficacy of SGB remains unclear.

Current evidence indicates that SGB can serve as a combined therapeutic approach, providing more significant clinical benefits to tinnitus patients (especially those with inadequate response to conventional treatments) with an acceptable overall safety profile. Therefore, it is recommended that clinicians integrate SGB into the comprehensive management strategy for tinnitus.

Future large scale, methodologically rigorous multi-center randomized controlled trials are still needed to standardize treatment protocols, assess cost effectiveness, and conduct long-term follow-up, in order to further clarify the optimal target population for SGB, refine treatment procedures, and establish its precise role in the clinical pathway for tinnitus.

## Conclusion

Our meta-analysis indicates that combining SGB with conventional therapy may be superior to conventional therapy alone in improving the effective treatment rate for tinnitus, enhancing cerebral blood flow velocity, and reducing tinnitus and anxiety-related distress scores.

However, the certainty of this evidence is moderate to low due to methodological limitations in the included trials, primarily the difficulty in implementing participant blinding owing to the invasive nature of SGB and incomplete reporting of follow-up data. Consequently, these findings should be interpreted with caution. Future rigorously designed, large-scale, multicenter randomized controlled trials with standardized protocols and extended follow-up are warranted to confirm the long-term efficacy and safety of this combination approach and to clarify its optimal role in tinnitus management.

## Data Availability

The datasets presented in this study can be found in online repositories. The names of the repository/repositories and accession number(s) can be found in the article/Supplementary material.
