# Supplementary material for: Efficacy and safety of stellate ganglion block for tinnitus: a systematic review and meta-analysis
Source: Front Neurol. 2026 Jan 20;17:1766506. doi: 10.3389/fneur.2026.1766506 (PMC12864091; doi:10.3389/fneur.2026.1766506)
Supplement: Supplementary file 1 [file Data_Sheet_1.docx]

Supplementary materials

[Supplement_1._Search strategy 1](#_Toc182689041)

[Supplement_2._Subgroup analysis by SGB session times and ultrasound guidance 3](#_Toc182689042)

[Supplement_3._Outcome Measures Used in the Included Studies 4](#_Toc182689042)

#

# Supplement 1. search strategy

| Pubmed | | |
| --- | --- | --- |
| #1 | ( "Stellate Ganglion"[Mesh] OR ("stellate ganglion"[tiab] AND (block*[tiab] OR inject*[tiab] OR inj[tiab] OR surg*[tiab] OR anesth*[tiab] OR neural*[tiab] OR denervat*[tiab] OR ablation[tiab])) OR "Cervicothoracic Ganglion"[Mesh] OR "cervicothoracic ganglion"[tiab] OR stellate ganglion block[tiab] OR stellate block[tiab] OR SGB[tiab] OR ((cervical[tiab] OR cervico-thoracic[tiab]) AND (sympathetic block[tiab] OR ganglion block[tiab]))) | 4544 |
| #2 | ( "Tinnitus"[Mesh] OR tinnitus[tiab] OR "ringing in the ears"[tiab] OR "ear ringing"[tiab] OR "head noise"[tiab] OR "subjective tinnitus"[tiab] OR "phantom sound"[tiab]) | 18177 |
| #3 | #1 AND #2 | 21 |
| Web of sciences | | |
| #1 | (TS=("stellate ganglion" NEAR/2 (block* OR inject* OR anesth*)) OR TS=("cervicothoracic ganglion" NEAR/2 (block* OR inject*)) OR TS=("stellate ganglion block") OR TS=("stellate block") OR TS=("stellate ganglion injection") OR TS=(SGB) OR TI=("stellate ganglion block")) | 1587 |
| #2 | ( TS=(tinnitus) OR TS=("ringing in the ears") OR TS=("ear noise*") OR TS=("phantom sound*") OR TS=("subjective tinnitus") OR TI=(tinnitus)) | 8944 |
| #3 | #1 AND #2 | 4 |
| Embase |  |  |
| #1 | ( 'stellate ganglion block'/exp OR 'stellate ganglion' OR 'cervicothoracic ganglion'/exp OR 'cervicothoracic ganglion' OR 'stellate ganglion block':ti,ab,kw OR 'stellate ganglion injection':ti,ab,kw OR 'cervicothoracic ganglion block':ti,ab,kw OR 'cervicothoracic ganglion injection':ti,ab,kw OR (stellate NEAR/3 ganglion NEAR/3 (block* OR inject* OR anesth*)):ti,ab,kw OR sgb:ti,ab,kw OR 'sympathetic block':ti,ab,kw) | 8203 |
| #2 | 'tinnitus'/exp OR 'tinnitus' OR tinnitus:ti,ab,kw OR 'ringing in the ears':ti,ab,kw OR 'subjective tinnitus':ti,ab,kw OR 'phantom sound':ti,ab,kw OR 'phantom noise':ti,ab,kw OR 'ear noise':ti,ab,kw OR 'head noise':ti,ab,kw OR ((ear* NEAR/3 (ringing OR noise)):ti,ab,kw) | 35941 |
| #3 | #1 AND #2 | 62 |
| Ovid | | |
| #1 | exp Stellate Ganglion/ | 2840 |
| #2 | (stellate ganglion block or stellate ganglion injection or SGB).ti,ab,kw. | 2720 |
| #3 | #1 or #2 | 4806 |
| #4 | exp Tinnitus/ | 10250 |
| #5 | (tinnitus or "ringing in the ears" or subjective tinnitus).ti,ab,kw. | 21519 |
| #6 | #4 or #5 | 23168 |
| #7 | #3 or #6 | 19 |
| Cochrane Library | | |
| #1 | MeSH descriptor: [Stellate Ganglion] explode all trees | 135 |
| #2 | ("stellate ganglion block"):ti,ab,kw | 560 |
| #3 | ("stellate ganglion injection"):ti,ab,kw | 118 |
| #4 | SGB | 351 |
| #5 | #1 or #2 or #3 or #4 | 702 |
| #6 | MeSH descriptor: [Tinnitus] explode all trees | 943 |
| #7 | (tinnitus):ti,ab,kw OR (ringing in the ears):ti,ab,kw OR (subjective tinnitus):ti,ab,kw OR (phantom sound):ti,ab,kw OR (phantom sound):ti,ab,kw | 3309 |
| #8 | #6 or #7 | 3309 |
| #9 | #5 and #8 | 7 |
| CNKI | | |
| #1 | SU=('星状神经节阻滞' + '颈胸神经节阻滞' + '星状神经节注射' + 'SGB' + 'sgb') OR TI=('星状神经节阻滞' + '颈胸神经节阻滞' + '星状神经节注射' + 'SGB' + 'sgb') OR FT=('星状神经节阻滞' + '颈胸神经节阻滞' + '星状神经节注射' + 'SGB' + 'sgb') | 16922 |
| #2 | SU=('耳鸣' + '主观性耳鸣' + '神经性耳鸣' + '颅鸣' + '脑鸣') OR TI=('耳鸣' + '主观性耳鸣' + '神经性耳鸣' + '颅鸣' + '脑鸣') OR FT=('耳鸣' + '主观性耳鸣' + '神经性耳鸣' + '颅鸣' + '脑鸣') | 189566 |
| #3 | #1 AND #2 | 38 |
| VIP | | |
|  | M=(星状神经节阻滞 + 颈胸神经节阻滞 + 星状神经节注射 + SGB + sgb ) AND M=(耳鸣 + 主观性耳鸣 + 神经性耳鸣+ 颅鸣 + 脑鸣) | 26 |
| Wangfang | | |
|  | (主题:(星状神经节阻滞 or 颈胸神经节阻滞 or 星状神经节注射 or SGB or sgb) or 题名或关键词:(星状神经节阻滞 or 颈胸神经节阻滞 or 星状神经节注射 or SGB or sgb)) and （主题:(耳鸣 or 神经性耳鸣 or 主观性耳鸣 or 脑鸣 or 颅鸣) or 题名或关键词:(耳鸣 or 神经性耳鸣 or 主观性耳鸣 or 脑鸣 or 颅鸣)） and (耳鸣 or 神经性耳鸣 or 主观性耳鸣 or 脑鸣 or 颅鸣)) | 68 |
| Sinomed | | |
| #1 | "星状神经节"[标题:智能] OR "星状神经节阻滞"[标题:智能] OR "星状神经节注射"[标题:智能] OR "颈胸神经节"[标题:智能] OR "SGB"[标题:智能] OR "sgb"[标题:智能] OR "星状神经节"[摘要:智能] OR "星状神经节注射"[摘要:智能] OR "SGB"[摘要:智能] | 6584 |
| #2 | "耳鸣"[标题:智能] OR "神经性耳鸣"[标题:智能] OR "主观性耳鸣"[标题:智能] OR "脑鸣"[标题:智能] OR "颅鸣"[标题:智能] OR "耳鸣"[摘要:智能] OR "神经性耳鸣"[摘要:智能] OR "主观性耳鸣"[摘要:智能] OR "脑鸣"[摘要:智能] | 29334 |
| #3 | #1 AND #2 | 68 |

**Supplement 2. Subgroup analysis by SGB session times and ultrasound guidance**


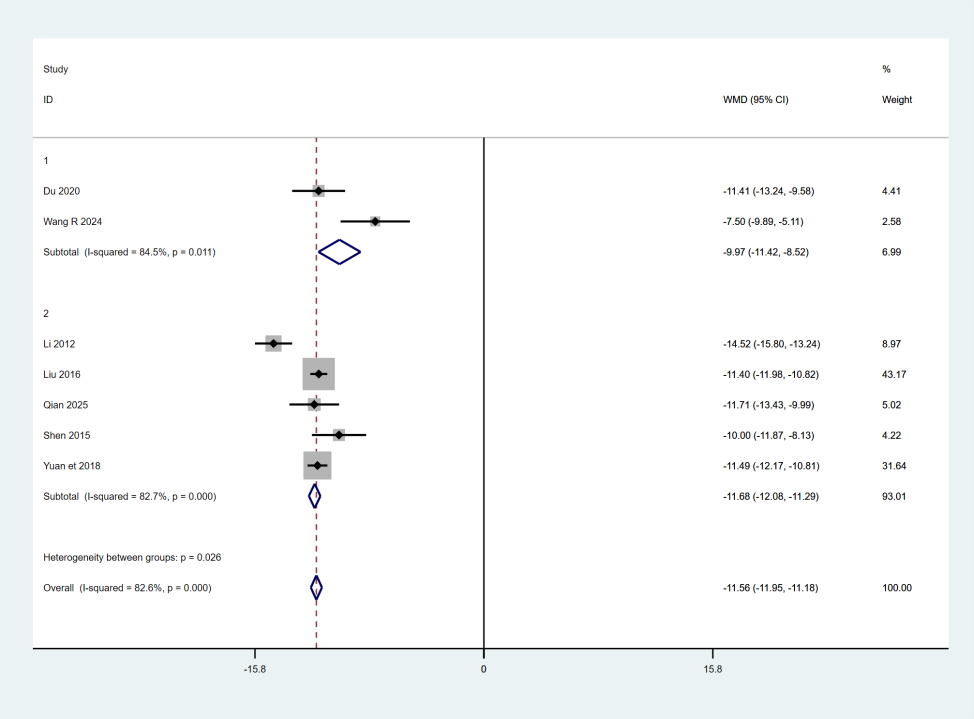


**Subgroup analysis by SGB session number**

**
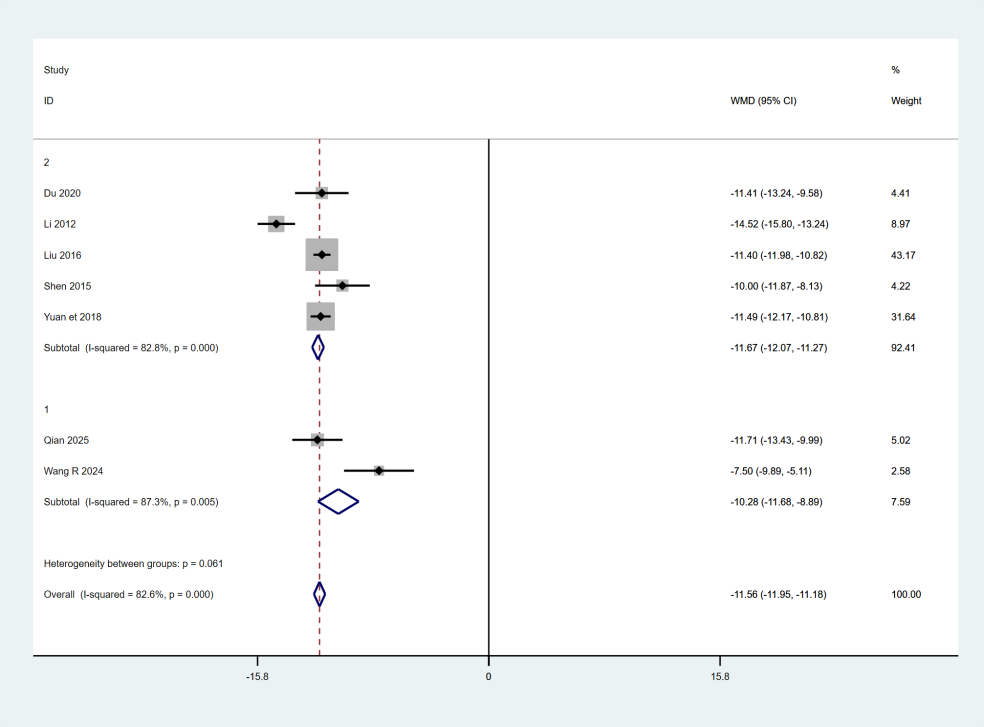
**

**Subgroup analysis by SGB ultrasound guidance**

**Supplement 3. Outcome Measures Used in the Included Studies**

| Qian Xxiaolan 2025 | Grundfast KM 2023 |
| --- | --- |
| Du Guoping 2020 | Liu Peng 2004 |
| Li Juanhong 2012 | Liu Peng 2008 |
| Wang Bing 2024 | Liu Peng 2004 |
| Wang Ruilin 2024 | Liu Peng 2004 |
| Wang Xufeng 2019 | Liu Peng 2008 |
| Yuan Jinghe 2018 | Liu Peng 2008 |
| Shen Min 2015 | Liu Peng 2008 |
| Liu Haipeng 2016 | Liu Peng 2008 |
| Yuan Jinghe et 2018 | Liu Peng 2008 |
| Xie Yi 2022 | NA |
| Qian Xxiaolan 2025 | Grundfast KM 2023 |

Liu Peng 2004:Liu P. Discussion on the grading of tinnitus severity and criteria for efficacy evaluation. Chinese Journal of Otorhinolaryngology in Integrative Medicine. 2004;(4):181-183. DOI: 10.16542/j.cnki.issn.1007-4856.2004.04.005.

Liu Peng 2008:Liu P, Li M. Reflections on the evaluation of tinnitus treatment efficacy. Chinese Journal of Otorhinolaryngology Head and Neck Surgery. 2008;43(9):710-713.doi:

10.3321/j.issn:1673-0860.2008.09.020

Grundfast KM 2023:Grundfast KM, Jamil TL. Evaluation and Management of Tinnitus: Are There Opportunities for Improvement?. Otolaryngol Head Neck Surg. 2023;168(1):45-58. doi:10.1177/01945998221088286
