# Supplementary material for: Efficacy and safety of stellate ganglion block for tinnitus: a systematic review and meta-analysis
Source: Front Neurol. 2026 Jan 20;17:1766506. doi: 10.3389/fneur.2026.1766506 (PMC12864091; doi:10.3389/fneur.2026.1766506)
Supplement: Supplementary file 2 [file Data_Sheet_2.docx]

| **Section and Topic** | **Item #** | **Checklist item** | **Location where item is reported** |
| --- | --- | --- | --- |
| **TITLE** | | |  |
| Title | 1 | Efficacy and Safety of Stellate Ganglion Block for Tinnitus: A SystematicReview and Meta-analysis | Title |
| **ABSTRACT** | | |  |
| Abstract | 2 | The structured abstract includes purpose,methods,results and Keywords. | Abstract: purpose |
| **INTRODUCTION** | | |  |
| Rationale | 3 | Describe in the introduction. | Background |
| Objectives | 4 | Describe in the introduction. | Background |
| **METHODS** | | |  |
| Eligibility criteria | 5 | Stated in the Methods-study selection. | Inclusion Criteria |
| Information sources | 6 | Stated in the Methods-search strategy. | Search strategy |
| Search strategy | 7 | Stated in the Methods-search strategy. | Search strategy |
| Selection process | 8 | Stated in the Methods-study selection. | Data Collection |
| Data collection process | 9 | Stated in the Methods-data extraction. | Data Collection |
| Data items | 10a | Describe in the Methods-outcome measurement. | Data Collection |
|  | 10b | Stated in the Methods-data extraction. | Data Collection |
| Study risk of bias assessment | 11 | Describe in the Methods-Quality assessment. | Quality Assessment |
| Effect measures | 12 | Describe in the Methods-data synthesis. | Statistical Analysis |
| Synthesis methods | 13a | All studies were eligible for the same synthesis. | / |
|  | 13b | Not required. | / |
|  | 13c | Not required. | / |
|  | 13d | Describe in the Methods-assessment of heterogeneity and publication bias. | Quality Assessment |
|  | 13e | Describe in the Methods-subgroups and sensitivity analyses. | Statistical Analysis |
|  | 13f | Describe in the Methods-subgroups and sensitivity analyses. | Statistical Analysis |
| Reporting bias assessment | 14 | Describe in the Methods-assessment of heterogeneity and publication bias. | Statistical Analysis |
| Certainty assessment | 15 | Stated in the Methods-quality of evidence. | Quality Assessment |
| **RESULTS** | | |  |
| Study selection | 16a | Stated in the Results-study selection. | Search Results |
|  | 16b | Stated in the Results-study selection. | Search Results |
| Study characteristics | 17 | Stated in the Results-characteristics of included studies. | Characteristics of the included studies |
| Risk of bias in studies | 18 | Stated in the Results-Risk-of-bias assessment. | Quality Assessment |
| Results of individual studies | 19 | Stated in the Meta-analysis Results-Total effective rate,SAS、THI、Vs、Vd、PSV、EDV, Adverse Reactions. | Total effective rate |
| Results of syntheses | 20a | Stated in the Meta-analysis Results-Total effective rate, SAS、THI. | Total effective rate,THI Score  ,SAS Score |
|  | 20b | Stated in the Meta-analysis Results Vs、Vd、PSV、EDV. | Basilar Artery Blood Flow Velocity,Carotid Artery Blood Flow Velocity |
|  | 20c | Stated in the Meta-analysis Results Adverse Reactions. | Adverse Reactions |
|  | 20d | Stated in the Meta-analysis Results Adverse Reactions. | Adverse Reactions |
| Reporting biases | 21 | Stated in the Meta-analysis Results-publication bias and sensitivity analysis. | Publication Bias Analysis,  Subgroups analysis and Sensitivity analysis |
| Certainty of evidence | 22 | Describe in the Results-quality of evidence. | / |
| **DISCUSSION** | | |  |
| Discussion | 23a | Describe in the discussion. | Discussion |
|  | 23b | Describe in the discussion. | Discussion |
|  | 23c | Describe in the discussion. | Discussion |
|  | 23d | Describe in the points for clinical practice and questions for future research. | Discussion |
| **OTHER INFORMATION** | | |  |
| Registration and protocol | 24a | The protocol of the review has been registered on PROSPERO(registration number: CRD420251242113) . | Systematic review registration |
|  | 24b | This systematic review and meta-analysis protocol has been published elsewhere. | / |
|  | 24c | No correction. | / |
| Support | 25 | This study is supported by 2 projects(Grant No. 2024WSJK125,KJQN202302709). | Funding |
| Competing interests | 26 | No competing interests. | Conflict of Interest |
| Availability of data, code and other materials | 27 | Not required. | / |

*From:*  Page MJ, McKenzie JE, Bossuyt PM, Boutron I, Hoffmann TC, Mulrow CD, et al. The PRISMA 2020 statement: an updated guideline for reporting systematic reviews. BMJ 2021;372:n71. doi: 10.1136/bmj.n71

For more information, visit: <http://www.prisma-statement.org/>
